# Surgical Training and Standardised Management Guidelines Improved the 30-Day Complication Rate After Abdominoplasty for Massive Weight Loss

**DOI:** 10.1007/s00268-017-4341-8

**Published:** 2017-11-28

**Authors:** E. Swedenhammar, B. Stark, A. Hedén Hållstrand, M. Ehrström, J. Gahm

**Affiliations:** 1 Department of Molecular Medicine and Surgery, Karolinska Institute, Karolinska vägen, 171 76 Stockholm, Sweden; 2 Department of Surgery, Capio S:t Görans Hospital, Stockholm, Sweden; 3 Department of Plastic and Reconstructive Surgery, Karolinska University Hospital, Solna, Sweden; 4 Capio Dalen Geriatric Hospital, Stockholm, Sweden

## Abstract

**Background:**

An increasing number of patients need reconstructive surgery after massive weight loss. The hypothesis was that surgical experience together with standardised management guidelines significantly decreases early complication rates after abdominoplasty for massive weight loss. The primary aim was to assess the 30-day complication rate after abdominoplasty following increased surgical training and experience. The secondary aim was to assess whether optimised management guidelines have an impact on the complication rate and patient safety.

**Methods:**

The outcome of 69 consecutive abdominoplasties operated by surgeons in 2011 (Group A) and 70 consecutive patients operated by plastic surgeons in 2010–2012 (Group B) was compared. Another Group of 70 consecutive patients operated by surgeons in 2013–2014 (Group C) was assessed since standardised guidelines for pre- and post-operative treatments and refinement of surgical technique had been introduced. The same surgeons participated in operations of Groups A and C. *χ* ^2^-test and Fisher’s exact test were applied to dichotomous data. Logistic regression test and ANOVA were used.

**Results:**

Group C had more comorbidities and was significantly older. 48 patients in Group A (70%), 31 in Group B (44%) and 13 patients in Group C (19%) had early complications. A significantly decreased rate of complications occurred with improved guidelines and surgical training and experience. (A vs. C *p* < 0.001 and A vs. B *p* = 0.008).

**Conclusions:**

Our results indicate that the rate of early complications after abdominoplasty for massive weight loss can be significantly reduced with improved surgical experience and standardised management guidelines.

Registered at Clinical Trial.gov (ID: NCT02679391).

## Introduction

Bariatric surgery is possibly the most effective way to obtain sustainable weight loss when BMI exceeds 40 [1, 2]. Massive weight loss leaves the patient with excessive soft tissue at various locations including the abdomen [3]. About 75–82% of post-bariatric surgical patients wish to undergo body contouring surgery in Sweden, and some of these operations are performed in general surgical units [4–7].

Body contouring surgery is associated with a high risk for surgical complications [8]. A report from the Swedish government states the importance of high volume of surgical procedure per surgeon and centralisation to fewer hospitals to decrease the level of complications in highly specialised care [9]. There is no clear data if this also applies to abdominoplasty.

The hypothesis of this study was that surgical training and experience together with standardised management guidelines significantly decrease early complication rates after abdominoplasty for massive weight loss. The primary aim of this study was to assess the 30-day complication rate after abdominoplasty for massive weight loss following increased surgical training and experience. The secondary aim was to assess whether optimised management guidelines have an impact on the complication rate and patient safety. The aesthetic outcome was not investigated in this study.

This study was approved by the Regional Ethics Review Board in Stockholm (D. nr. 2012/1997-31/4, 2015/2017-32). Registered at Clinical Trial.gov (ID: NCT02679391).

## Materials and methods

### Patients

**Fig. 1:**
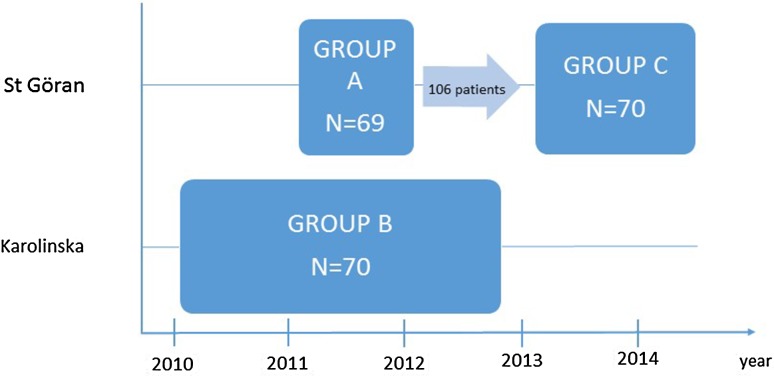
Flowchart of patients included in the study. Group A from 13th of May to 19th of December 2011. Group B from 14th of January 2010 to 7th of December 2012. Group C from 22th of January 2013 to 3th of June 2014. 106 consecutive patients were operated between Groups A and C. Patients were referred from bariatric centres and general practitioners

This was a retrospective cohort study. All patients included in the study were admitted for surgery based on the Swedish national criteria for massive weight loss [10] and first assessed at the Department of Reconstructive Plastic Surgery, Karolinska University Hospital, Stockholm, Sweden (Table 1), the allocation of patients only depending of the waiting list at Karolinska Hospital. The cohorts of patients were not biased regarding age, BMI or other criteria. The timeline for those assessed is shown in Fig. 1. Patients were found by searching medical records for the national operation codes for abdominoplasty (QBJ00–QBJ99). The patients were operated either at the Department of Reconstructive Plastic Surgery, Karolinska University Hospital or at the Department of General Surgery, Capio S:t Görans Hospital, Stockholm. At S:t Görans Hospital each procedure was performed by any two of four specialists in general surgery. The four general surgeons at St Görans Hospital had a special interest to start performing abdominoplasties. During the first (less than 10) operations in Group A, a plastic surgeon supervised and educated the general surgeons in a systemic manner.

**Table 1:** Patients included and excluded in accordance with national guidelines for abdominoplasty after massive weight loss^10^

Indications	Contraindications
Abdominal skin ptosis ≥ 3 cm	Weight instability
Weight stability ≥ 6 months	Smoking
Smoke-free ≥ 6 weeks prior to surgery	BMI > 35
BMI ≤ 28 or a significant weight reduction equivalent to 80% of excess weight	Psychological imbalance or non-treated psychiatric disease
	Advanced cardiovascular disease

At the Department of Reconstructive Plastic Surgery, each procedure was performed by any of 15 specialists in plastic surgery although not always with specific training in body contouring surgery. Records were retrieved from the first 69 consecutive patients operated by the general surgeons at Capio S:t Görans Hospital, Stockholm, Sweden (Group A) from 13th of May to 19th of December 2011. Records from 70 consecutive patients operated from 14th of January 2010 to 7th of December 2012 by plastic surgeons at the Department of Plastic and Reconstructive Surgery, Karolinska University Hospital, Stockholm, Sweden (Group B) were assessed. Due to waiting time and operation queues at Karolinska University Hospital, the period for Group B was considerably lengthier. All information was collected by the authors ES and AH.

Two years after, the general surgeons started to perform abdominoplasties at St Görans Hospital records from another 70 consecutive patients were evaluated, between 22th of January 2013 to 3rd of June 2014 (Group C). Between Group A and *C,* the pre-, peri- and post-operative care guidelines had been standardised and implemented (Table 2), thus the starting date for Group C. All patients in Group C were operated by the same four general surgeons, and the outcome was compared with those of Groups B and A. One hundred and six consecutive patients (not investigated in this study) were operated by the same group of general surgeons between the end of Group A and beginning of Group C. Patient demographics are shown in Table 3. A period of 4–6 weeks of non-smoking was recommended before surgery. The unknown number of smokers could have been higher, and it was, therefore, difficult to comment on the impact of smoking on the results. Follow-up assessments in all groups were performed after one week by a nurse, and after three months by a senior surgeon.

**Table 2:** Guidelines for patients in Group C

Guidelines	
Medication	Paracetamol 665 mg × 3, 1 week
	Oxycodone slow release 10 mg × 2, 1 week
	Oxycodone 5 mg when needed
	Tranexamic acid 1 g × 3 for 1 day, but not if there is a drop in Haemoglobin over 30 g/L
	Antibiotics: isoxazolpenicillin 2 g pre-operative or clindamycin 600 mg
	Low molecular-weight heparin, 2500–5000IE × 1, 10 days post-operative
Mobilization	Early mobilisation, as soon as possible after surgery
	Firm girdle 4 weeks
	Underwear girdle for 3 months
Controls	Haemoglobin: pre-operative and every 8 h post-operatively Day 1 and thereafter once a day
	MEWS (Modified Early Warning Score): once every 4 h Day 1 post-operatively

**Table 3:** Descriptive statistics for patient Groups A, B and C

	A (*n* = 69)	B (*n* = 70)	C (*n* = 70)	*p* value overall test
Female, *n* (%)	66 (95.7%)	60 (85.7%)	63 (90%)	0.136
Age, mean (sd)	41.4 (9.5)	38.6 (11.37)	46.8 (10.07)**	<0.001
BMI before surgery, mean (sd)	26 (1.91)	26.3 (2.16)	26^a^ (1.69)	0.596
Skin ptosis (cm), mean (sd)	4.4 (1.51)	4.4^b^ (1.3)	4.2 (1.09)	0.473
Weight loss in BMI units, mean (sd)	17.8^c^ (5.12)	17.4^c^ (4.8)	16.3^c^ (5.11)	0.236
ASA 1, *n* (%)	45 (65.2%)	46 (65.7%)	32 (45.7%)*	0.0332
ASA 2, *n* (%)	24 (34.8%)	22 (31.4%)	36 (51.4%)*	
ASA 3, *n* (%)	0 (0%)	2 (2.9%)	2 (2.9%)	
*Diabetes*				
Type I, *n* (%)	1 (1.4%)	0 (0%)	1 (1.4%)	0.368
Type II, *n* (%)	2 (2.9%)	1 (1.4%)	7 (10%)	0.040
Former smokers	10 (14.5%)	15 (21.4%)	26 (37.1%)	0.01

*p* values for overall tests are presented. If *p* < 0.05, pairwise comparisons for A versus B and A versus C are calculated and presented as *p < 0.05, **p < 0.01 and ***p < 0.001

Smokers are presented as former smokers, but the charts did not always clearly state (in Group C) whether the habit had ceased or not

^a^Information missing for one patient in Group C

^b^Information missing for nine patients in Group B

^c^Information missing for three patients in Group A, two patients in Group B and three patients in Group C

### Surgery

A team of 2 general surgeons in Groups A and C and 2 plastic surgeons in Group B performed each operation (Table 4). The same group of surgeons participated in surgery on Groups A and C. Some plastic surgeons performed liposuction before the abdominoplasty in Group B. When appropriate a panniculectomy was performed [11, 12]. An abdominal rectus diastasis (ARD) wider than 3 cm was repaired with a double row of absorbable, self-retaining barbed suture, PDO 2/0. When detected intraoperatively, midline and incisional hernias were repaired with double row sutures. Post-operative drains were used in all patients in Groups A and B, but varied in Group C depending on the surgeon’s preference.

**Table 4:** Operation data for patient Groups A, B and C presented as number and proportion (%) or median (Md)

	A (*n* = 69)	B (*n* = 70)	C (*n* = 70)	*p* value overall test
Abdominoplasty	69 (100%)	67 (96%)	70 (100%)	0.108
Hernia repair	1 (1%)	7 (10%)	5 (7%)	0.088
Plication of ARD	3 (4%)	25 (36%)***	5 (7%)	<0.001
T-incision	2 (3%)	4 (6%)	1 (1%)	0.411
Resected tissue (g), Md (95% CI)	1685 (1538.6–1943.6)	1370.5^a^ (1205.2–1554.9)	1524 (1338.6–1718.5)	0.053
Liposuction	0 (0%)	10 (14%)**	0 (0%)	<0.001
Drain-bilateral	69 (100%)	70 (100%)	24 (34%)***	<0.001

*p* values from overall tests are presented. If *p* < 0.05, pairwise comparisons for A versus B and A versus C are calculated and presented as *p < 0.05, **p < 0.01 or ***p < 0.001

^a^Information missing for four patients in Group A and two patients in Group B

### Complications

Early 30-day complications were defined as minor or major. Wound infection, seroma and small wound dehiscence were defined as minor whereas post-operative bleeding, tissue necrosis, thromboembolism, revision surgery and systemic events requiring intensive care treatment were considered major complications. For better comparison, a Clavien Dindo (C–D) table was created [13]. Wound infection (C–D class II) was defined by the administration of oral or intravenous antibiotics secondary to local or systemic signs of infection. Seroma (C–D class I) was only reported when it was described in the notes either as a swollen abdomen 1 week after surgery or when aspirated (C–D class IIIa).

### Standardised management guidelines in Group C

Guidelines applied to Group C included the administration of tranexamic acid, intravenous antibiotics, and focused on optimal perioperative and post-operative care (Table 2). By the time Group C was investigated, all patients had received the same perioperative and post-operative care, and a more well-defined surgical technique used, including meticulous haemostasis of the perforator vessels and less extensive dissection of the upper part of the abdominal wall. Post-operative care was standardised in Group C (Table 2). There were no equivalent guidelines in Group A or B.

## Statistics

*χ* ^2^-test and Fisher’s exact test were applied to dichotomous data. The demographic data of the patients were expressed as median and standard deviations. Logistic regression test and ANOVA were used. Since there were three groups, ANOVA was used to check if overall differences between the groups were significant, and if so each variable was tested A versus B and A versus C. To avoid the risk of random errors, B versus C was not calculated. There was no randomisation of patients in the three cohorts, and any difference calculated could have been the result of patients systematically differing in one or more variables. To avoid this bias, a logistic regression analysis adjusting for age, BMI and ASA-class were completed. Since many variables had a low prevalence, separate models were not estimated for each complication. The model used estimated the occurrence of (1) at least one minor complication, (2) at least one major complication and (3) no complication. The statistics was calculated by R Software for Mac version 3.2.2.

## Results

### Patients

Prior to surgery the 3 groups were similar in terms of mean BMI and mean skin ptosis. Patients in Group C were significantly older with more comorbidity than those in Groups A and B. Group C had a significantly higher prevalence of ASA II (*p* = 0.027, Group A vs. C) (Table 3).

### Surgery and medication

More extensive surgery took place in Group B (Table 4). There was a significant difference in pre-, intra- and post-operative medications between all three groups (Table 5). One dose of intravenous or oral antibiotics was administrated, isoxazolylpencillin or, if allergic, a lincosamide, at least 30 min prior to surgery. Eleven patients (16%) received one dose of intravenous antibiotics in Group A, compared to 27 (39%) in Group B (Group A vs. B, *p* = 0.004). Sixty-six patients (94%) in Group C received one dose of intravenous antibiotics (Group A vs. C, *p* < 0.001).

**Table 5:** Recommendations for prophylaxis treatment in patient Groups A, B and C presented as numbers and proportion (%)

	A (*n* = 69)	B (*n* = 70)	C (*n* = 70)	*p* value overall test
Antibiotics	11 (16%)	27 (39%)**	66 (94%)***	<0.001
Tranexamic acid	0 (0%)	9 (13%)**	65 (93%)***	<0.001

*p* values from overall tests are presented. If *p* < 0.05, pairwise comparisons for A versus B and A versus C are calculated and presented as *p < 0.05, **p < 0.01 or ***p < 0.001

No patient was given tranexamic acid in Group A, whereas 9 patients (13%) received tranexamic acid in Group B (Group A vs. B, *p* = 0.003), and 65 patients (93%) in Group C (Group A vs. C, *p* < 0.001). Low molecular weight heparin 10 days post-operatively (Fragmin^®^ 5000 IE or Klexane^®^ 100 mg/ml, 0.4 ml) was prescribed to all the patients in Group A (100%), to 37 patients (53%) in Group B (the length of treatment varying from 3 to 12 days) and to 63 of the patients (90%) in Group C.

### Complications

Complications are shown in Tables 6 and 7. 48 patients in Group A (70%), 31 in Group B (44%) and 13 patients in Group C (19%) had early complications. The difference between Groups A and C and between Groups A and B were statistically significant (*p* < 0.001 and *p* = 0.008, respectively).

**Table 6:** Complication data for patient Groups A, B and C presented as numbers and proportion (%)

	A (*n* = 69)	B (*n* = 70)	C (*n* = 70)	*p* value overall test
*Minor complication*				
Wound infection	32 (49%)^a^	19 (27%)*	7 (10%)***	<0.001
Seroma	8 (12%)	5 (7%)	6 (9%)	0.648
Wound dehiscence	5 (7%)	3 (4%)	1 (1%)	0.202
*Major complication*				
Bleeding	22 (32%)	7 (10%)**	5 (7%)***	<0.001
Transfusion	12 (17%)	3 (4%)*	0 (0%)***	<0.001
Tissue necrosis	2 (3%)	2 (3%)	0 (0%)	0.474
Thromboembolic event	0 (0%)	0 (0%)	0 (0%)	>0.999
Reoperation	3 (4%)	1 (1%)^b^	0 (0%)	0.130
Intensive care unit stay	3 (4%)	0 (0%)	0 (0%)	0.035
Patients with complications	48 (70%)^a^	31 (44%)^c^ **	13 (19%)***	<0.001

*p* values from overall tests are presented. If *p* < 0.05, pairwise comparisons for A versus B and A versus C are calculated and presented as *p < 0.05, **p < 0.01 or ***p < 0.001

^a^Information missing for four patients in Group A

^b^Information missing for one patient in Group B

^c^Information missing for four patients in Group A and one patient in Group B

**Table 7:** Complications classified in accordance with Clavien–Dindo

	A (*n* = 69)	B (*n* = 70)	C (*n* = 70)
I	7 (10%)	4 (6%)	5 (7%)
II	34 (49%)	19 (27%)	7 (10%)
IIIa	0 (0%)	4 (6%)	0 (0%)
IIIb	3 (4%)	1 (1%)^a^	0 (0%)
IV	3 (4%)	0 (0%)	0 (0%)
V	0 (0%)	0 (0%)	0 (0%)

The highest-ranking complication is registered for each patient. Groups A, B and C presented as numbers and proportion (%)

^a^Information missing for one patient in Group B

### Minor complications

Wound infection (Clavien–Dindo classification II) was most common in Group A, 32 patients (49%) compared to 19 patients in Group B (27%, *p* = 0.014 compared to Group A), and 7 patients in Group C (10%, *p* < 0.001 compared to group A). A seroma (C–D class I) occurred in 8 patients in Group A (12%), in 5 patients in Group B (7%) and in 6 patients in Group C (9%).

### Major complications

Bleeding (C–D class I) was significantly higher in Group A (22 patients, 32%) than in Group B *(*7 patients, *p* = 0.003) and Group C (A vs. C, *p* < 0.001). Significantly, more patients in Group A (12 patients, 17%) versus 3 in Group B (4%, *p* = 0.015) required post-operative blood transfusion (C–D class II). Two of the patients in Group A required intensive care for hypovolemic shock, and one patient needed intensive care due to over-dosage of opioids (C–D IVa). No thromboembolic events were seen in any of the three groups.

Univariate analysis showed an overall statistical difference between the cohorts for six of the variables. The results, shown in Table 8, showed a difference remaining between the cohorts when adjusted for the demographic variables. The odds of a minor or major complication occurring were higher in Group A compared to Groups B and C, respectively.

**Table 8:** OR (odds ratio) and 95% confidence interval estimated from logistic regression for outcome ≥ 1 minor complication, ≥ 1 major complication and no complication

Group	≥1 Minor complication (*n* = 204) Adjusted^a^ OR (95% CI)	≥1 Major complication (*n* = 207) Adjusted^a^ OR (95% CI)	No complication (*n* = 203) Adjusted^a^ OR (95% CI)
A	Ref	Ref	Ref
B	0.29 (0.132–0.601)	0.46 (0.209–0.993)	3.18 (1.538–6.769)
C	0.17 (0.069–0.376)	0.19 (0.067–0.467)	9.04 (4.059–21.368)

ORs are adjusted for age, BMI and ASA-class at the time of surgery

^a^The numbers do not add up to 209 patients due to information missing in all three groups, as shown in Table 6

## Discussion

Facing a growing population of massive weight loss patients and the high-reported incidence of early complications after body contouring surgery [8, 14], the aim was to assess if increased surgical experience and training in performing abdominoplasties combined with standardised management guidelines decreased 30-day complication rates after abdominoplasty. The frequency of complications in Group A was unacceptable and the reason that the general surgeons developed new management guidelines. The overall complication rates in Group B were in line with previously reported data [10, 14–17]. Previously reported studies indicate overall complication rates as high as 55.5%. Age, smoking, male gender, multiple procedures and body mass index are independent risk factors that increase the risk for complications [14–17]. In a recent retrospective study by Botero et al., similar results were seen when assessing 153 patient with body contouring surgery [17], with the larger part of complications defined as minor (C–D I–IIIa). In *i*, however, these were significantly higher despite more extensive surgery in Group B with comparable patient demographics. This possibly reflects a difference in plastic surgery training, i.e. experience of performing abdominoplasty, between the surgeons performing the procedures in Group A (General surgeons) and those in Group B (plastic surgeons) as discussed by Mioton et al. [18]. The four general surgeons had very small experience of abdominoplasties prior to Group A and improvement in complication rates between Groups A and C could partially be explained by improved surgical experience.

Between Groups A and C, improved pre-, intra- and post-operative management routines and checklists had been introduced (Tables 2, 5). Despite more comorbidity and higher ASA grades in Group C compared to Group A, fewer major complications were noted, which the regression analysis confirmed. One must take into account the team surrounding the patient at the general surgical ward and in the OR got more experienced and perhaps added to the results in Group C, but the management guidelines also helped. The improved surgical experience/technique of performing abdominoplasties appears to have had a substantial effect, significantly decreasing the overall complication rate. This is in line with international trends and experience in both highly specialised care and more routine surgery such as hernia repair, where high-volume surgery performed by fewer surgeons significantly increase overall survival and patient safety, and decrease complication rates [19].

Pre-operative antibiotic prophylaxis, as one dose pre-operatively, seems to lower the risk for wound infection in our study. Data on the usefulness of antibiotics are conflicting and consensus is lacking. The prophylactic use of antibiotics is advisable according to the Swedish Council on Health Technology Assessment, but evidence in the case of abdominoplasty is low [20]. Data providing convincing evidence on the effect of tranexamic acid on blood loss during reconstructive plastic and breast surgery are sparse [21–23]. The usage of both tranexamic acid and LMWH can seem contradictive, and the scientific evidence for this approach are sparse nevertheless, it is not unheard of [24].

The repair of the rectus diastasis differed between the groups. A diastasis wider than 3 centimetres is considered pathological [25] and plastic surgeons by tradition repair rectus diastasis perhaps in contrast to general surgeons. The use of drains was largely omitted in Group C in contrast to common routine. In a retrospective study from Rodby et al., it was shown that abdominoplasty with lesser undermining above the umbilicus, liposuction, and no post-operative drain resulted in fewer infections, and the seroma rate was in the lower range of the spectrum [26]. High-quality evidence-based reports with comparable groups and operative techniques are insufficient. The present study revealed that patients in all 3 groups had seroma and our data are in concordance with other reports with prevalence rates of 5–30% [27–30].

## Strengths of the study

Patients were operated consecutively in all three Groups. Group C was assessed when all management guideline improvements had been implemented. Statistical calculations were applied systematically and potential bias in interpretation was investigated with appropriate regression analysis.

## Weaknesses of the study

This is a retrospective cohort study. The lack of standardised criteria for some of the complications introduced a degree of subjectivity of assessment by the investigating surgeon at follow-up. The present study gives an overview of retrospective data and general conclusions can thus be questioned. A general registry for complications after abdominoplasty for massive weight loss does not exist, but could be a part of the existing registry for bariatric surgery in the future. The systematic registration of such data would certainly help to provide more statistically conclusive evidence.

## Conclusions

This study provides good statistical evidence for an observed trend in terms of improved surgical experience and training in performing abdominoplasties and standardised management guidelines leading to significant reductions in surgical complication rates and increased safety for the patient. Aesthetic outcome and patient satisfaction were not addressed in this study, but should be considered in future studies.
